# Large-Scale and Multiscale Networks in the Rodent Brain during Novelty Exploration

**DOI:** 10.1523/ENEURO.0494-20.2021

**Published:** 2021-05-08

**Authors:** Michael X Cohen, Bernhard Englitz, Arthur S. C. França

**Affiliations:** 1 Donders Centre for Medical Neuroscience, Radboud University Medical Center, 6525 GA, Nijmegen; 2 Computational Neuroscience Lab, Department of Neurophysiology, Donders Institute for Brain, Cognition and Behavior, Radboud University Nijmegen, 6525 XZ, Nijmegen The Netherlands

**Keywords:** cortex, eigendecomposition, local field potential, networks, oscillations, source separation

## Abstract

Neural activity is coordinated across multiple spatial and temporal scales, and these patterns of coordination are implicated in both healthy and impaired cognitive operations. However, empirical cross-scale investigations are relatively infrequent, because of limited data availability and to the difficulty of analyzing rich multivariate datasets. Here, we applied frequency-resolved multivariate source-separation analyses to characterize a large-scale dataset comprising spiking and local field potential (LFP) activity recorded simultaneously in three brain regions (prefrontal cortex, parietal cortex, hippocampus) in freely-moving mice. We identified a constellation of multidimensional, inter-regional networks across a range of frequencies (2–200 Hz). These networks were reproducible within animals across different recording sessions, but varied across different animals, suggesting individual variability in network architecture. The theta band (∼4–10 Hz) networks had several prominent features, including roughly equal contribution from all regions and strong inter-network synchronization. Overall, these findings demonstrate a multidimensional landscape of large-scale functional activations of cortical networks operating across multiple spatial, spectral, and temporal scales during open-field exploration.

## Significance Statement

Neural activity is synchronized over space, time, and frequency. To characterize the dynamics of large-scale networks spanning multiple brain regions, we recorded data from the prefrontal cortex, parietal cortex, and hippocampus in awake behaving mice and pooled data from spiking activity and local field potentials (LFPs) into one data matrix. Frequency-specific multivariate decomposition methods revealed a cornucopia of neural networks defined by coherent spatiotemporal patterns over time. These findings reveal a rich, dynamic, and multivariate landscape of large-scale neural activity patterns during foraging behavior.

## Introduction

Neural activity is coordinated across multiple spatial and temporal scales, ranging from spike-timing correlations across pairs of neurons (Gray et al., 1989) to resting-state fMRI networks (Gusnard et al., 2001), and from ultra-fast 600 Hz ω oscillations in primary sensory cortex (Timofeev and Bazhenov, 2005) to infra-slow fluctuations linked to 0.05-Hz oscillations in the gastric system (Richter et al., 2017). Coordinated activity is thought to allow for neural circuits to maximize communication efficiency, multiplex information, flexibly route information flow, and functionally bind cell assemblies (Singer, 2009; Jensen and Mazaheri, 2010; Wang, 2010).

However, most neuroscience investigations are limited to a single spatial scale [e.g., action potentials or local field potential (LFP)], and cross-scale investigations are often based on univariate or bivariate measures (e.g., coherence between action potentials from one neuron with the LFP recorded on the same or different electrode; Pesaran et al., 2018). Mass-univariate and mass-bivariate approaches have been crucial to the development of neuroscience, for example, understanding computational principles such as neural tuning (Hubel and Wiesel, 1959; Carandini, 2005; Hebart and Baker, 2018) and inter-regional synchronization (Fries et al., 2001). However, these approaches may obscure spatiotemporal patterns embedded across populations of neurons within and across brain regions (Kriegeskorte and Kievit, 2013; Cunningham and Yu, 2014; Ritchie et al., 2019; Williamson et al., 2019).

In contrast, multivariate data analysis methods have proven useful at identifying spatially distributed patterns that reflect lower-dimensional dynamics or that encode sensory representations or memories (Pang et al., 2016). Furthermore, correlational patterns may provide a “contextual activation” that shapes subsequent local computations (Cohen and Kohn, 2011; Priesemann et al., 2014; Kohn et al., 2016; Alishbayli et al., 2019).

Multivariate analyses are often used to identify “functional networks” in the brain. Network neuroscience is receiving growing attention in the literature (Bassett and Sporns, 2017; Bassett et al., 2020), because of its potential for revealing patterns and dynamics in the brain that might be inaccessible in univariate analyses. Although the term functional network does not have a specific and widely agreed-on definition (Power et al., 2011), we use that term to indicate a set of data channels that are combined in a way that maximizes their time series covariance patterns.

In the present study, a recently developed set of multivariate methods [generalized eigendecomposition (GED); Cohen, 2017] enabled us to discover multiscale, inter-regional functional networks during active behavior, by combining data from multiunits and LFPs. We found a salient, empirical grouping of the networks into a small number of frequency bands (average of 7). Within each frequency band, multiple subnetworks were both simultaneously and independently active. Some networks (e.g., in theta) were spatially distributed across the brain, while other networks (typically in higher frequencies) were more localized to one or two regions. Spiking activity contributed less systematically to brain-wide networks compared with LFP. The analyses revealed both idiosyncratic and reproducible network characteristics within-animals and across-animals, which suggests that the spatial organization of large-scale networks is subject to individual variability. Overall, our findings reveal a complex landscape of dynamic neural activity that spans multiple spatial, spectral, and temporal scales.

## Materials and Methods

### Data acquisition

Six male mice with Bl57/6jbackground (B6;129P2-Pvalbtm1(cr)Arbr/J or Ssttm2.1(cre)Zjh/J) between four and five months of age, weighing between 27 and 34 g, were used in this study. All experiments were approved by the Dutch central commission for animal research (Centrale Commissie Dierproeven) and implemented according to approved work protocols from the local University Medical Centre animal welfare body (approval number 2016-0079).

**Figure 1.**
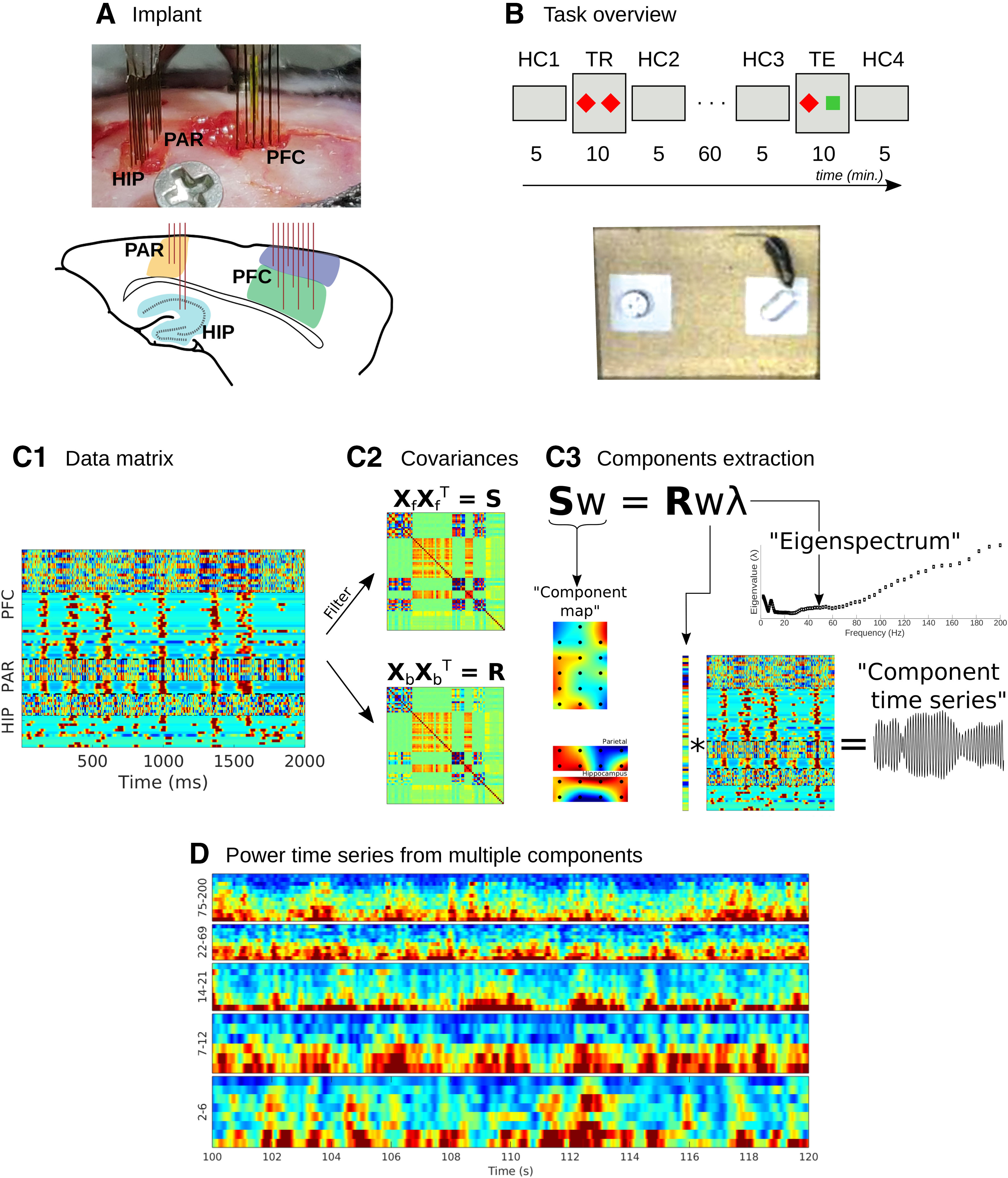
Overview of recording locations, task design, data analysis, and sample data. ***A***, 32-channel custom-designed electrode array (HIP: hippocampus; PAR: parietal cortex; PFC: prefrontal cortex). The line drawing underneath illustrates the approximate locations of the electrodes on a sagittal slice. ***B***, Task flow and timing (HC1-4: home cage sessions 1-4; TR: training; TE: testing). The red diamonds and green square indicate objects placed in the arena. The picture underneath is from a camera placed overhead. ***C1***, A data matrix with combined LFP and multiunits (smoothed with a 30-ms FWHM Gaussian) from three different regions. ***C2***, Data covariance matrices for the data snippet shown in ***C1***, either narrowband-filtered (S) or broadband (R). A generalized eigendecomposition of these two matrices (panel ***C3***) provides a set of eigenvectors (w) and corresponding eigenvalues (λ), from which three pieces of information are extracted: The component spatial map (the eigenvector multiplied by the covariance matrix), the component time series (the eigenvector multiplied by the data matrix), and the separability of narrowband vs. broadband activity (the eigenvalue for one frequency; the eigenvalues over frequencies creates an eigenspectrum). Illustrated here is one eigenvalue solution for one frequency; in practice, the number of solutions (w/λ pairs) corresponds to the number of data channels, and this entire procedure is repeated across a range of frequencies. ***D***, Multiple components can be isolated from each frequency, with distinct temporal dynamics. Example component power time series are illustrated from 20 seconds of a recording; each row corresponds to a distinct component. Frequency groups are based on empirical frequency boundaries (described later) and components are sorted within each frequency band based on total component energy.

Each animal was implanted with 32 electrodes divided into three regions of the brain (see Fig. 1*A*): 16 electrodes targeted to the prefrontal cortex [spread in the coordinates anterior-posterior (AP): 0.5 and 1.5; medial-lateral (ML): 0.25 and 0.75; in three columns of electrodes in different depths: 2.0, 1.5, and 1.0], eight electrodes targeted to the parietal cortex [AP: −2 and −2,25; ML: 1.0 and 1.75; dorsal-ventral (DV): 0.5], and eight electrodes targeted to the hippocampus (AP: −2 and −2,25; ML: 1.0 and 1.75; DV: 0.5). Interelectrode distance was 250 μm and typical impedances were between 0.1 and 0.9 MΩ. More details about how to build these kinds of custom-designed electrodes are presented elsewhere (França et al., 2020). A metal reference screw was placed on the skull over the cerebellum (AP: −5, ML: 1.0, DV: 0.5), which was lowered until contact with the cerebrospinal fluid but avoided contact with the superior sagittal sinus and inferior cerebellar vein. Offline, an average reference was computed for each brain region and subtracted from each electrode in the corresponding region.

Although the anatomic targets were identical in all animals, minor differences in implantation and in individual brain anatomy mean that the electrode recording tips may have been in slightly different cortical and hippocampal fields in different animals.

Animals were recorded in the sessions depicted in Figure 1*B*. The recording sessions alternated between their familiar home cage and an unfamiliar location that contained novel objects. In particular, each mouse went through the same succession of six experiment sessions. (1) Home cage recording of 5 min. (2) Training phase of 10 min, in which the animal was placed in an unfamiliar environment that contained two novel objects. (3) Home cage recording of 5 min. One hour then passed (in the home cage) with no recordings. (4) Home cage recording of 5 min. (5) Testing phase, in which the animal was returned to the unfamiliar environment that contained one object seen during the training phase and one novel object. (6) Home cage recording of 5 min. Mice were connected via electrode fibers to the data acquisition board via a cable that hung from top of the Faraday cage, but were otherwise unrestrained. There was no particular task or objective that was trained, nor were any rewards provided.

Mice tended to explore the objects for brief periods of time (hundreds of milliseconds to seconds), whereas our data analysis approach used longer windows for temporal filtering and averaging to ensure high signal-to-noise quality. We therefore focused on possible state changes across the different task sessions, as opposed to time-locking to the on/offsets of transient object exploration periods.

LFP data were down-sampled to 1000 Hz. Excessively noisy channels, determined based on visual inspection, were removed (0–4 per recording session; average of 1.2). Independent components analysis (ICA) was run using the eeglab toolbox (Delorme and Makeig, 2004) and the jade algorithm (joint approximation diagonalization of eigen-matrices), which defines components by maximizing kurtosis (the fourth order statistical moment used to index non-Gaussianity; Cardoso, 1999). Components clearly identifiable as non-neural origins were projected out of the data. Non-physiological noise components are characterized by sharp transients or slow deflections that are usually several orders of magnitude larger than the neural dynamics, and are therefore identified by visual inspection using the data viewer in eeglab. We removed, on average 1.9 (range: 0–5) components per dataset, out a maximum of 32. The recording sessions began and ended with some contact with the experimenter, and we therefore excluded the first and last 10 s of each recording session to exclude possible artifacts and neural activity patterns associated with being handled or moved into or out of the box.

Data and MATLAB analysis code are available at https://data.donders.ru.nl/collections/di/dcmn/DSC_4546_462.

### Spike-sorting and multiunit extraction

The raw (30 kHz) voltage recordings were regional-average-referenced to eliminate possible volume-conduction artifacts, and were then filtered between 300 and 6000 Hz using a zero phase-shift FIR1 filter kernel. Spike-sorting was done for each electrode separately given the interelectrode spacing of 250 μm, which makes it unlikely to observe the same neuron on multiple electrodes. Indeed, we did not find excessive correlations across units from different electrodes (see Extended Data Fig. 1-1 for an example between-unit correlation matrix).

10.1523/ENEURO.0494-20.2021.f1-1 Extended Data Figure 1-1 Left plot shows an example multiunit correlation matrix from one recording session. The right plot shows a histogram of all unique off-diagonal correlation values. These plots illustrate that our spike-sorting approach was not overly contaminated by identifying the same units on multiple channels. Download Figure 1-1, PDF file.

Because our goal here was to obtain information about neural spiking activity as it related to the population and to LFP dynamics, rather than evaluating tuning properties of individual neurons, we chose an automatic spike-sorting approach that separated multiunits from noise or artifacts (Trautmann et al., 2019). We therefore term these signals “multiunit” to indicate that the resulting time series may reflect a mixture of action potentials from multiple neurons.

Multiunits were extracted via a general-purpose spike-sorting suite (*autoSort*, available via our open code repository https://bitbucket.org/benglitz/controller-dnp/src/master/Access/SpikeSorting/), implemented in MATLAB. Briefly, *autoSort* performs the following sequence of steps to achieve automatic and unbiased sorting of neural signals:

- Candidate spike waveforms (“spikes”) were detected based on a negative threshold of 4 SDs of the background noise (estimated as 1.48 times the median absolute deviation, to avoid artifacts that inflate the SD).
- Candidate spikes were then aligned to their minimum after the trigger and cut out within a window of [–0.7,1.2] ms relative to the alignment time.
- Principal components analysis (PCA) was performed on a random subset of spikes (*N*_S_ = 5000 per recording) to estimate a projector to a six-dimensional subspace that retained most of the variance in the data.
- Hierarchical clustering (based on *Ward* distance) with a set maximal number of clusters (*N*_C_ = 3) was performed on this representation, and all spikes beyond the *N*_S_ selection were assigned to these clusters on the basis of their Euclidean distance to the cluster centers.
- Clusters were then *post hoc* automatically selected and fused on the basis of the shape and similarity between their average waveforms, i.e. (1) clusters were excluded if they had no significantly positive “hump” after the negative alignment peak, if they had a significantly positive peak before the negative alignment peak, or if the waveform was longer or larger than expected for an extracellular spike; and (2) clusters were fused if the correlation and Euclidean distance between their average waveforms were above or below preset thresholds, respectively.

These steps and criteria led to an extraction of 0–2 multiunits per electrode. The average rate of spikes per second from all animals and recordings was 13.2 (SD 5.9, minimum 0.07, maximum 51.6). A binary spike time series was constructed for each multiunit, and smoothed with a 30-ms full-width at half-maximum Gaussian to create a continuous signal. This continuous signal was entered into the data matrix as one channel (Fig. 1*C*).

### Frequency-specific components using GED

We followed existing procedures for extracting multivariate components that have been detailed and validated in several previous publications, based on the mathematical framework of GED. Using ground-truth simulations, it has been shown that GED is more accurate and robust to noise compared with other common multivariate methods such as PCA and ICA (Tomé, 2006; Nikulin et al., 2011; de Cheveigné and Parra, 2014; Cohen, 2017). A brief overview of the analysis procedure is provided here.

The goal is to identify a spatial filter that provides a scalar weight for each data channel (LFP and multiunits) such that the weighted sum of narrowband-filtered channel time series is maximally different from the broadband channel time series. The method is based on data covariance matrices because they contain all pairwise linear relationships, making the method multivariate. As described below, two covariance matrices are compared, one matrix (**R**) based on the broadband (non-temporally filtered) data, and one matrix based on the narrowband filtered data (**S**).

Channel-by-channel covariance matrices were created by multiplying the mean-centered data matrices by their transpose. To increase covariance stability, we cut the continuous data into a series of non-overlapping 2-s segments, and computed the covariance matrix of each segment. The even-numbered epochs were used to create the **S** (signal) covariance matrix and the odd-numbered epochs were used to create the **R** (reference) covariance matrix. This was done to have non-identical data across the two matrices. After computing covariance matrices for each segment (there were around 70 segments in the home cage sessions and 140 segments in the training/testing sessions), the average covariance matrices **S** and **R** were computed across segments. Euclidean distance from each individual covariance matrix to the average was computed (this is equivalent to the Frobenius norm of the matrix difference), and any segments with a distance >3 SDs from the average were excluded, and the final covariance matrix was re-computed without the outliers. On average, 0.85% of covariance matrices were excluded per analysis (range: 0–3%).

To create the spatial filter per frequency, we start from maximizing the Rayleigh quotient:

(1) $$W_{\max}^{}{=}_{\text{ w}}^{\arg\text{ max}}\frac{W^{T}SW}{W^{T}\text{R}W},$$

where **S** and **R** are channel covariance matrices obtained from the narrowband filtered data and the broadband data, respectively (Fig. 1*C*). One can think of Equation 1 as a multivariate signal-to-noise ratio, and the goal is to find a channel vector **w** that maximizes this ratio. The solution comes from a generalized eigenvalue decomposition on the two matrices:

(2) SW=RWΛ.

The diagonal matrix **Λ** contains the eigenvalues, each of which is the ratio of Equation 1 for the corresponding column of **W**, which is a matrix in which the columns are the eigenvectors. Thus, we obtain *m* spatial filters for an *m*-channel dataset. The solutions are linearly independent from each other, though they are not constrained to be orthogonal as with PCA (this is because eigenvector orthogonality is guaranteed only for symmetric matrices, and **R**^−1^**S** is non-symmetric). Equation 2 is repeated for a range of temporal frequencies (see below), each using a different **S** matrix (the covariance matrix created from narrowband filtered data) with the same **R** matrix.

A small amount of shrinkage regularization (1%) was applied to the **R** matrix to improve the quality of the decomposition (Lotte and Guan, 2011). In our experience, 1% shrinkage has no appreciable effect on decompositions of clean, full-rank, and easily separable data, and considerably improves the decompositions of noisy or reduced-rank data. In Equation 3 below, γ is the amount of shrinkage (0.01, corresponding to 1%), α is the average of all eigenvalues of **R**, and **I** is the identity matrix:

(3) $$\tilde{R}=\left(1-\gamma \right)R+\gamma \alpha I.$$

In Results, we refer to each spatial filter as a “component,” and when speculating on the interpretation of these components, we use the term “network” to indicate that each component reflects a combination of data channels that maximizes a covariance pattern, which is consistent with the idea of a functional network (Bassett and Sporns, 2017; Power et al., 2011). The component time series was obtained by multiplying **w** by the channels-by-time data matrix (this is how the eigenvector acts as a spatial filter). For all signals, any time series values exceeding 4 SDs from the mean of the time series were excluded, which reduced the possibility of residual non-representative data from influencing the results. The component map was obtained by multiplying **w** by the **S** covariance matrix (Haufe et al., 2014).

The component map is anatomically interpretable as the projection of the spatial filter. However, the eigenvectors **w** have higher spatial frequency characteristics because they invert volume conduction and suppress irrelevant channels. We therefore used the correlations of eigenvectors across frequencies to define empirical frequency bands (Cohen, 2021). This was implemented by identifying clusters in the matrix of squared correlations across the top eigenvector from all frequencies using the dbscan algorithm. Unlike some clustering methods such as k-means or hierarchical clustering, dbscan does not necessarily assign each frequency to a cluster. Thus, clusters are formed only if strong correlations are present, and frequencies without strong intercorrelations are left unclustered. As shown in Figure 3, this grouping was quite salient in the data. After identifying empirical frequency boundaries within each recording session, a subsequent k-means clustering was performed to identify consistencies in frequency boundaries across sessions and animals.

**Figure 2.**
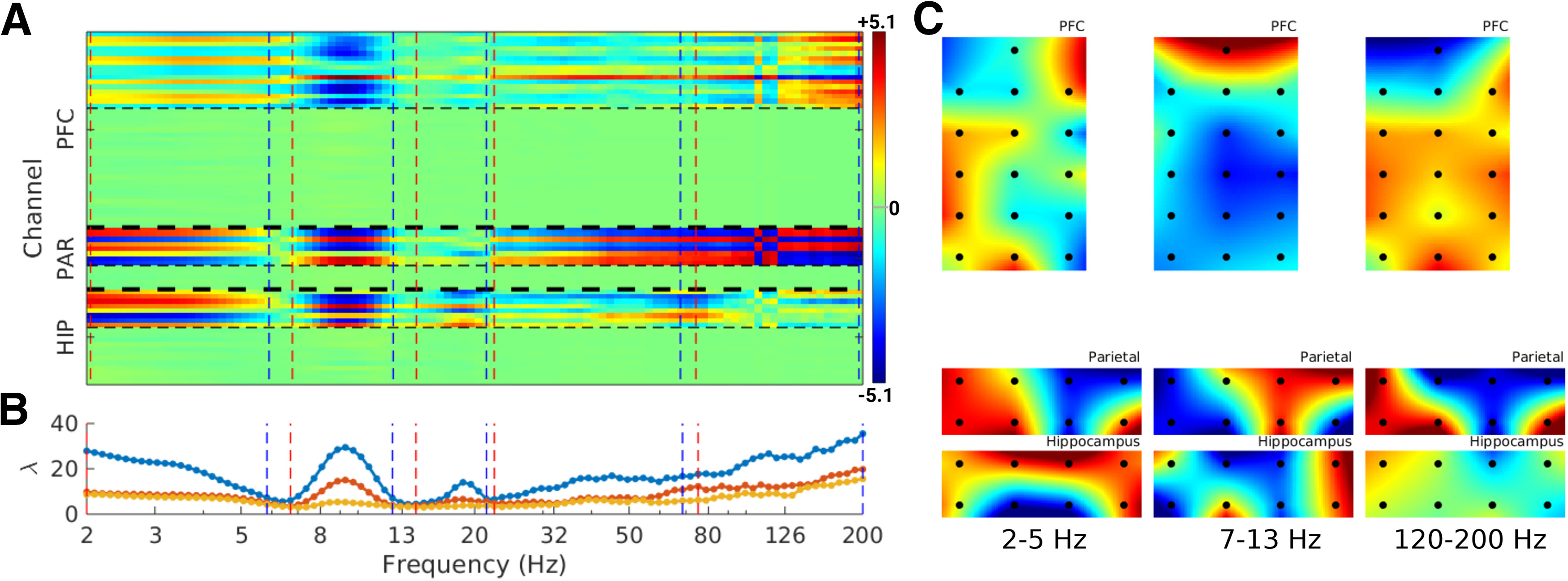
Generalized Eigendecomposition enables spectrally resolved source separation across 3 areas for a single recording. ***A***, Spatial maps over all three regions per frequency (each column corresponds to one frequency). The thick horizontal dashed lines show inter-regional boundaries, and thin horizontal dashed lines show within-region boundaries between LFP (top) and multiunit (bottom) channels. Within-region rows are ordered according to the channel index in the dataset, not according to anatomical location. The colors indicate the strength of the contribution of that channel to the brain-wide component (data were per-frequency normalized prior to GED, so the color values are comparable across frequencies), vertical dashed lines show the empirically defined frequency boundaries (detailed later): red lines indicate the lower bounds of the frequency band and blue lines indicate the upper bounds. ***B***, Eigenspectra from the largest three components per frequency, which highlights that there can be multiple separable components at the same frequency. The map in panel A is only for the top eigenspectrum (blue line). ***C***, Example topographical maps of the anatomical distribution of the filter projections for the indicated frequency ranges. Each black dot is the location of an electrode. In all columns, medial is to the left and anterior is to the top.

The entire procedure described above was repeated independently for each animal, experiment session, and filtering frequency. This allowed us to examine the reproducibility of the components both within and across animals.

Data were temporally narrowband filtered by convolution with a Morlet wavelet, defined here as a Gaussian in the frequency domain (Cohen, 2019). Extracted frequencies ranged from 2 to 200 Hz in 100 logarithmically spaced steps. The full-width at half-maximum of the Gaussian varied from 2 to 5 Hz with increasing frequency. The multiunit channels were not narrowband filtered (they were already smoothed with a 30-ms Gaussian). Any large-scale spike-field coherence patterns would manifest as cross-channel terms in the frequency-specific covariance matrices.

We computed a “region bias score” to determine whether the components were driven by one region or whether all regions contributed to the component. This was quantified as the square root of the average of the squared eigenvector elements per region. That produced a three-element vector, which we normalized to sum to 1. The region bias score was defined as the Euclidean distance between this empirical vector and an “ideal shared region” vector of [1 1 1]/3. The idea is that if all brain regions have average eigenvector components that are equal in magnitude, then that vector will be close to [1 1 1]/3, and thus the empirical distance to the ideal vector will approach zero. As one or two regions start to dominate the component, the normalized average eigenvector elements vector (e.g., producing an empirical vector of [0.6 0.3 0.1]) will move further away from the ideal vector. The maximum possible distance is 1.

Subspace dimensionality was computed via permutation testing. The ability to derive inferential statistical values is one of the important advantages of GED over descriptive decompositions such as PCA or ICA. The idea here was to generate a distribution of maximal eigenvalues that could be expected under the null hypothesis that **S** and **R** contain the same information (note from Eq. 1 that the expected eigenvalue under the null hypothesis is 1, but maximum eigenvalues could be larger because of sampling variability). In the real data, each 2-s data segment has two covariance matrices: one from the narrowband filtered signal and one from the broadband signal. To generate null-hypothesis eigenvalues, we randomly assigned each covariance matrix to average into the **S** or **R** covariance matrices. GED was performed and the largest eigenvalue was stored. This procedure (randomizing covariance matrices into **S** or **R** and storing the largest eigenvalue) was repeated 200 times for each frequency. Finally, the maximum of the largest eigenvalues was taken as the most extreme eigenvalue that can be expected under the null hypothesis that there are no differences between the **S** and **R** matrices (per frequency). The number of actual eigenvalues (from the analysis without shuffling) above this extreme H0 value was taken as the dimensionality of the subspace. Note that this permutation method accounts for multiple comparisons over M components because it selects the most extreme value of M components on each iteration. Cleaning the covariance matrices via Euclidean distances was performed during permutation testing as described above.

Entropy was computed for each data channel using k = 40 bins for discretization:

(4) $$H=\sum_{i=1}^{k}y_{i}\log_{2}y_{i}.$$

Finally, within-frequency, intercomponent phase synchronization was computed via the weighted phase-lag index (Vinck et al., 2011), which is a modification of phase synchronization designed to remove any possible artifacts of volume conduction. This was important for our analyses because all networks were derived by different weightings of the same channels, and because the separate components at the same frequency were not constrained to orthogonality.

### Distribution shape via kurtosis

Non-Gaussianity is considered an indicator of an information-rich signal. This comes from the central limit theorem, which leads to the assumption that random noise, and random linear mixtures of signals, will produce Gaussian distributions. We therefore quantified the kurtosis (4th statistical moment of a distribution; the kurtosis of a pure Gaussian distribution is 3) as a measure of the non-Gaussianity of the component time series. We computed kurtosis for the narrowband filtered signal and its amplitude envelope at each component.

Component time series kurtosis was computed as the 4th statistical moment of the component time series. We extracted kurtosis from both the real part of the narrowband signal and the amplitude envelope (extracted via the Hilbert transform). The amplitude envelope had overall higher kurtosis (Extended Data https://doi.org/10.1523/ENEURO.0494-20.2021.f2-1), which is not surprising considering that amplitude is a strictly non-negative quantity.

Nearly all frequencies had kurtosis higher than 3, indicating leptokurtic distributions characterized by narrow peaks and fatter tails. This is consistent with suggestions that brain activity is characterized by extreme events and long-tailed distributions (Buzsáki and Mizuseki, 2014). Curiously, all six animals exhibited a dip in kurtosis in the theta band (∼9 Hz) (Extended Data https://doi.org/10.1523/ENEURO.0494-20.2021.f2-1B), indicating a platykurtic distribution with data values clustered towards zero and relatively fewer data points having extreme values (the tails of the distributions) (Extended data https://doi.org/10.1523/ENEURO.0494-20.2021.f2-1C). This may be related to the known sawtooth-like shape of hippocampal theta (Scheffer-Teixeira and Tort, 2016.

Note that unlike independent components analysis, GED is based purely on the signal covariance (second moment) and not on any higher-order statistical moments. Thus, non-Gaussian distributions are not trivially imposed by the decomposition method, but instead arose from the data without bias or selection. 

## Results

### Data matrices and narrowband source separation

We created channels X time data matrices with 50–80 channels per animal (28–32 LFP channels plus all detected multiunits; Fig. 1), and applied a dimensionality-reduction and guided source-separation method that isolates features of the data that maximally separate narrowband from broadband activity based on GED of covariance matrices (Cohen, 2017). GED was applied after narrowband filtering the data from 2 to 200 Hz in 100 logarithmically spaced steps, producing a succession of narrowband components. Each component is a weighted average of channels that maximizes energy at that frequency. There are multiple components per frequency that were sorted according to their eigenvalue, which encodes the separability between the narrowband and broadband energy.

Figure 2 illustrates results from one example recording session. This example highlights several consistent features that are expanded on later, including (1) different frequencies engage different electrodes across different regions; (2) some frequencies (e.g., theta) recruit multiregional networks whereas other frequencies preferentially engage one or two regions; (3) large-scale networks were dominated by LFP whereas multiunits made relatively little (though significant) contributions; (4) the local regional referencing ensured that the components reflected the coordination of multiple local dipoles (seen as the balance between blue and red colors in the map) instead of long-range volume-conducted fields. The components time series had non-Gaussian distributions, indicative of true signals rather than noise, which is expected to be Gaussian-distributed (Extended Data Fig. 2-1).

**Figure 3.**
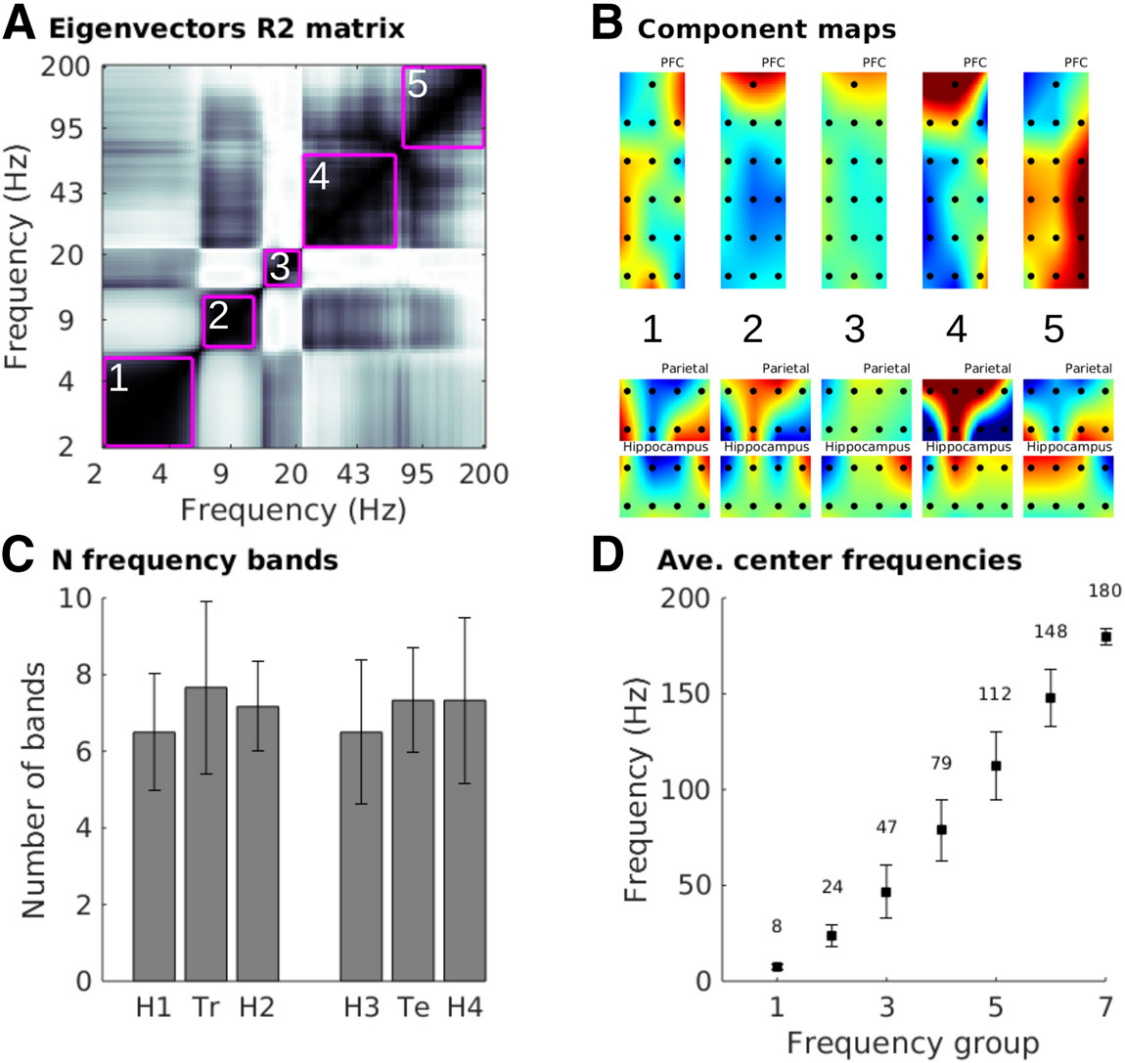
Distinct frequency bands separate clearly in the LFP data with specific spectrotemporal profiles. ***A***, R2 correlation matrix across all pairs of frequency-specific eigenvectors, with pink boxes drawn around empirically derived clusters (based on the dbscan algorithm), from one recording. The cluster boundaries separate spatially distinct topographies across different frequency ranges. ***B***, Topographical maps of the spatial filter from the frequency bands in panel A. White/black numbers indicate corresponding bands/maps. ***C***, Aggregated results of the number of empirical frequency bands per experiment session (H1-4 indicate home sessions; Tr indicates training session; Te indicates test session). Error bars show standard deviations across the six animals. ***D***, Center frequencies for each group as defined by k-means clustering analysis over animals. Error bars show standard deviations across 100 repeats of the k-means clustering algorithm with different random initializations, and the numbers above each data point shows the average center frequency from that band.

10.1523/ENEURO.0494-20.2021.f2-1 Extended Data Figure 2-1 Kurtosis, a measure of non-Gaussianity of a distribution (see text below), computed on frequency-specific component time series. The red and blue lines in panel ***A*** show kurtosis per frequency for the narrowband-filtered time series (blue) and amplitude envelope (red), averaged over all animals and sessions. The horizontal dashed line indicates the expected kurtosis of a pure Gaussian distribution. ***B***, Kurtosis over frequencies for each animal separately. Note the striking decrease in kurtosis in the theta band in all animals. ***C***, Example time series histograms illustrating the platykurtic effect at 8 and 11 Hz for two different animals and sessions.
*Distribution shape via kurtosis*. Non-Gaussianity is considered an indicator of an information-rich signal. This comes from the central limit theorem, which leads to the assumption that random noise, and random linear mixtures of signals, will produce Gaussian distributions. We therefore quantified the kurtosis (4th statistical moment of a distribution; the kurtosis of a pure Gaussian distribution is 3) as a measure of the non-Gaussianity of the component time series. We computed kurtosis for the narrowband filtered signal and its amplitude envelope at each component.
Component time series kurtosis was computed as the 4th statistical moment of the component time series. We extracted kurtosis from both the real part of the narrowband signal and the amplitude envelope (extracted via the Hilbert transform). The amplitude envelope had overall higher kurtosis (Extended Data Fig. 2-1), which is not surprising considering that amplitude is a strictly non-negative quantity.
Nearly all frequencies had kurtosis higher than 3, indicating leptokurtic distributions characterized by narrow peaks and fatter tails. This is consistent with suggestions that brain activity is characterized by extreme events and long-tailed distributions (Buzsáki and Mizuseki, 2014). Curiously, all six animals exhibited a dip in kurtosis in the theta band (∼9 Hz; Extended Data Fig. 2-1*B*), indicating a platykurtic distribution with data values clustered towards zero and relatively fewer data points having extreme values (the tails of the distributions; Extended Data Fig. 2-1*C*). This may be related to the known sawtooth-like shape of hippocampal theta (Scheffer-Teixeira and Tort, 2016).
Note that unlike ICA, GED is based purely on the signal covariance (second moment) and not on any higher-order statistical moments. Thus, non-Gaussian distributions are not trivially imposed by the decomposition method, but instead arose from the data without bias or selection. Download Figure 2-1, DOCX file.

### Empirically derived frequency bands

Electrophysiology data are often grouped into frequency bands according to integer boundaries (e.g., 4–10 Hz), which may miss, artificially separate, or artificially combine the rhythms naturally occurring in the brain. We therefore applied a recently established method (gedBounds) to derive empirical frequency bands based on the definition of a “frequency band” as a range of frequencies that have highly correlated spatiotemporal dynamics (Cohen, 2021). GedBounds works by clustering the matrix of squared correlations across the eigenvectors from all frequencies (Fig. 3*A*). It is a purely data-driven alternative to labeling frequencies based on a priori expectations.

This analysis revealed an average of seven bands in the range of 2–200 Hz (Fig. 3*B*). The number of frequency bands was not significantly different between experiment sessions (one-way ANOVA, *F*_(5,25)_ = 0.45). Average center frequencies were computed by k-means clustering on the empirical frequencies. Because k-means can produce different clusters on each run, we re-seeded the clustering 100 times. The average cluster center frequencies, along with their SDs, are shown in Figure 3*D*. The dbscan algorithm used to identify clusters within each dataset groups frequencies together only when strong correlations are present (Cohen, 2021), and there is no constraint that neighboring frequencies belong to the same cluster. Thus, the consistency in number of bands, and the boundaries of those bands, across sessions and animals is not a trivial result of forcing each frequency to belong to its neighbor’s cluster.

These results show that grouping electrophysiology time series into spectral bands has an empirical basis and is not arbitrary or an artifact imposed by narrowband filtering. The empirically derived frequency ranges varied over animals and task sessions, and were not systematically affected by the task session. However, we treated frequency as a continuous variable in subsequent analyses rather than grouping into discrete bins.

### Component reproducibility

The anatomic targets of the electrode implants were identical in all animals. However, individual variability in functional organization can mean that the GED patterns are idiosyncratic and thus different across animals. Likewise, if the spatiotemporal patterns that GED isolates reflect stable features of the brain, then the patterns should be highly similar in different experiment sessions within the same animal. On the other hand, it is possible that the spatiotemporal patterns are dynamic and are more affected by cognitive factors than by individual differences.

**Figure 4.**
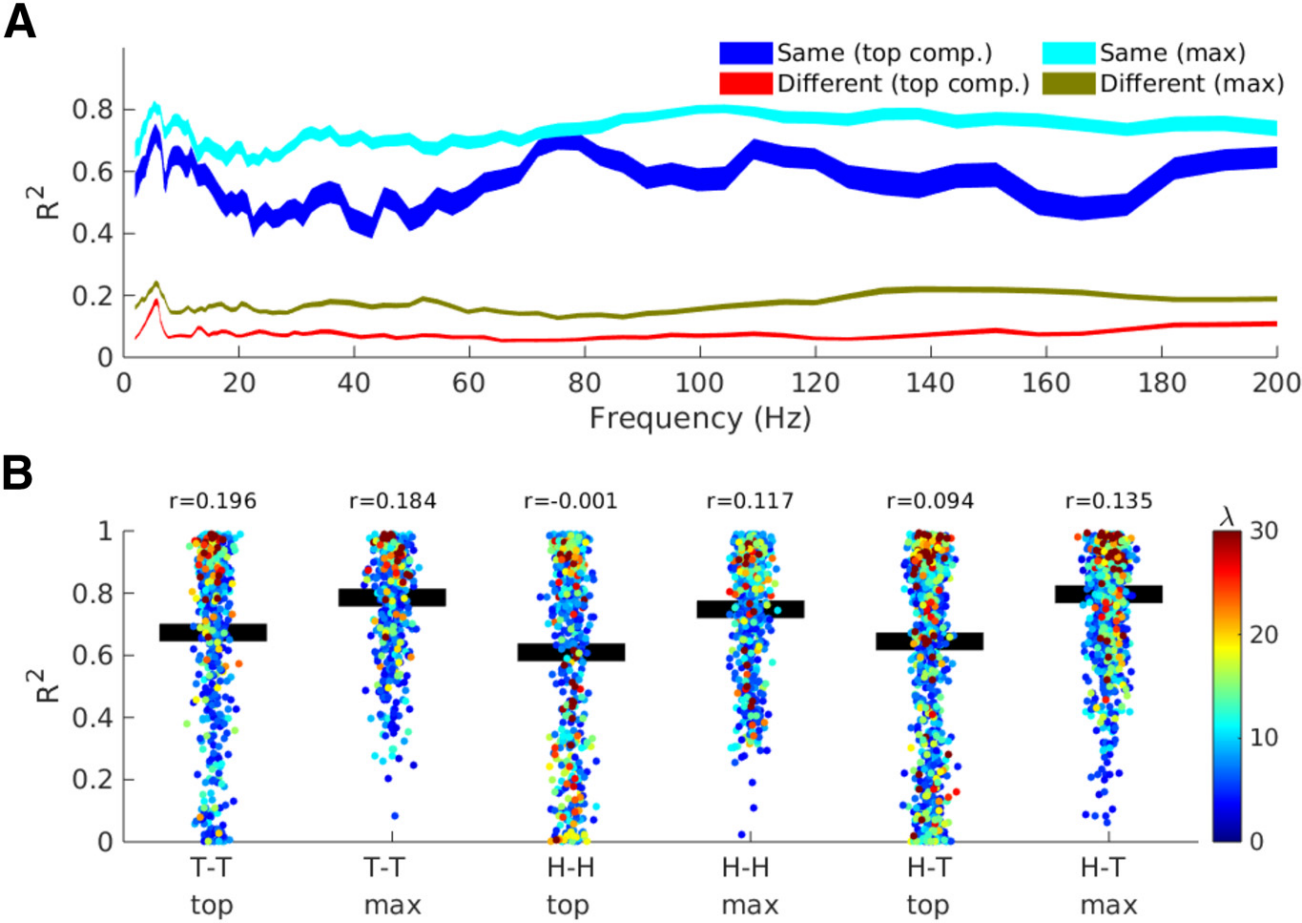
Component topographies are reproducible within animals in different sessions, yet differ across animals. ***A***, R2 spatial correlations per frequency. The analysis was run on the components with the largest eigenvalue per frequency (“top comp.”), and by selecting the largest correlation amongst the top two components (“max”). ***B***, Each individual correlation, separated according to the experiment sessions from which the spatial map pairs were drawn (“T-T” indicates train-test pairs, “H-H” indicates home-home pairs). Black bars indicate the mean R2. The color of each dot is the average of the eigenvalues of the component pair (which indicates the separability of the narrowband from broadband signals), and the r-value on top of each column is the correlation between the spatial map R2 and the average eigenvalue.

To address questions about component map reliability, we measured map reproducibility, quantified as spatial correlations, both across experiment sessions within each animal, and in the same session across animals. When pooling across all experiment sessions, we observed robust within-animal component topographies (*R*^2^ spatial correlations in the range of 0.4–0.8 over the frequency spectrum; see Fig. 4*A*). In contrast, spatial correlations across animals were low, with averaged *R*^2^ values below 0.2. Because the decompositions were performed on the data from each session independently, this pattern of results indicates that (1) the components were stable within each animal over different sessions (over the course of the ∼2-h recording), and that (2) component maps are idiosyncratic, with different spatial patterns in different animals.

The spatial correlations described above were done using only the component with the largest eigenvalue for each session and each frequency. It is possible that the same neurophysiological network was identified as “component 1” in one experiment session and “component 2” in a different session. We therefore modified the correlation analysis to compute the four unique correlations across the top two components from each session/frequency, and stored only the largest correlation coefficient. Although this selection procedure is biased because we selected the strongest correlation out of a set, the same bias was applied within-animals and across-animals. The correlations were overall stronger, but the conclusion is the same as when correlating only the top components: spatiotemporal patterns were stable within animals, and variable across animals.

We next assessed whether the maps were modulated by the different experiment sessions by separating *R*^2^ values according to experiment session. Figure 4*B*, scatter plots, shows all frequencies (each dot is an animal-frequency pair), but we averaged frequencies together for the statistics because Figure 4*A* indicates comparable relationships across the frequency domain. We then tested the correlation coefficients in a one-way ANOVA with the factors train-test, home-home, and train/test-home. In other words, we tested whether the maps were more similar to each other when the animals were in a similar experiment context. However, this effect was not statistically significant (*F*_(2,10)_ = 2.17, *p* = 0.16).

Inspection of the distribution of *R*^2^ values in Figure 4*B* show considerable spread of the correlations, which was only partially resolved by selecting the maximum correlation of the top two components. We suspected that at least some of this variation could be because of the separability of the components from broadband. “Separability” in a GED analysis is quantified as the eigenvalue, which is the multivariate ratio between the narrowband from the broadband covariance matrices along the direction of the eigenvector. We therefore correlated the *R*^2^ values with the average of the eigenvalues of each component-pair. Most correlations between map-similarity and eigenvalue were in the range of 0.1–0.2. Thus, it appears that, to some extent, the narrowband components that are better separated from the background spectrum are more likely to be stable over time.

### Region specificity of components

**Figure 5.**
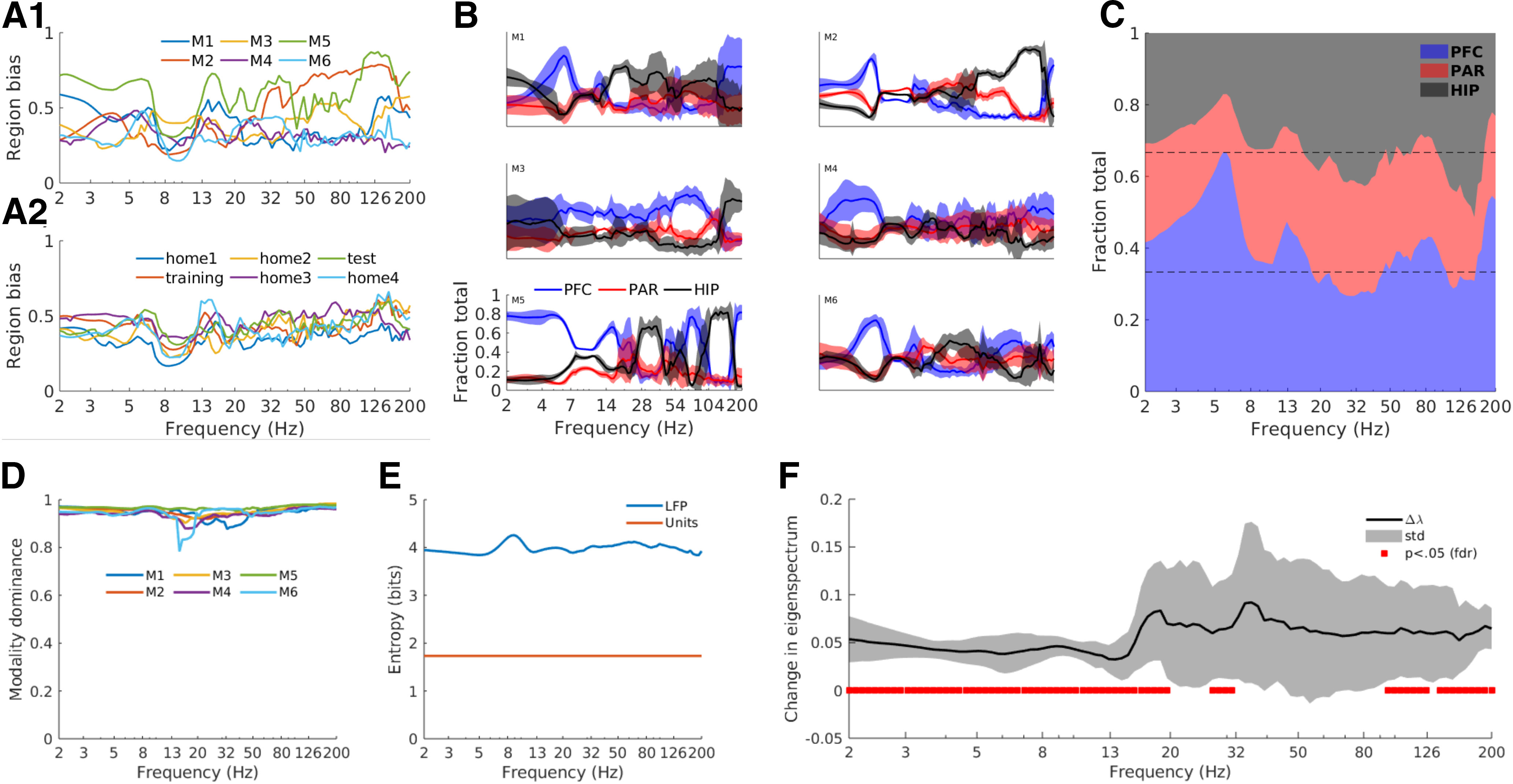
All recorded regions contributed to the components per frequency, with some frequencies showing regional dominance. ***A***, The region bias index for each animal (***A1***) and averaged over animals for each experiment session (***A2***). Values close to 0 indicate equal spread of components across all three brain regions, whereas values close to 1 indicate that a single region dominates the component. ***B***, The fraction of total component energy attributable to each region, normalized to the sum over all three regions (thus, the sum per frequency is 1). Each panel is a different animal, averaged over experiment sessions. Patches indicate one standard deviation above and below the mean across sessions, which illustrates the reproducibility of these characteristics over time (six sessions spanning 2 hours). All panels have the same tick marks and axis labels as the lower-left panel. The group-average regional fractions are shown in panel ***C***. Horizontal lines at 1/3 and 2/3 indicate equal contribution of all three regions to the component. ***D***, The modality dominance spectrum quantitatively showed that components were predominantly driven by LFP instead of by multiunits. ***E***, Entropy spectrum shows that LFP channels had higher entropy compared to the multiunits (multiunits’ entropy is the same for all frequencies). ***F***, The multiunits made significant contributions to the components over most frequencies except in the range of 20-90 Hz. Positive values indicate better separability when multiunits are included. The black line is the average over all animals, and the surrounding patch indicates one standard deviation around that average. Red lines show significant changes relative to zero at p<.05, FDR corrected for multiple comparisons over frequencies.

Given that our data matrices included signals from three brain regions, we next determined whether the components truly reflected inter-regional temporally coherent networks, or whether they were driven by a single region. This was assessed through a regional bias score, in which a score of zero indicates exactly equal contributions from all three regions, whereas a bias score of one indicates that the component is driven entirely by one region with no contributions from the other two regions (Fig. 5*A*).

The bias scores were mostly between 0.4 and 0.6 within each animal (Fig. 5*A*), indicating that all three regions contributed to the components to varying degrees. The frequency range that stood out was theta, which exhibited a notable dip in the bias score. Thus, all three brain regions contributed to large-scale networks in the theta range.

This bias score is an aggregate measure; we next investigated the contributions of each region to each frequency, separately for each animal. Figure 5*B* shows both diversity and commonalities in the regional contributions across the different animals. In these plots, overlapping lines at *y* = 1/3 indicates that all three regions contributed equally to the components, whereas regional dominance is reflected by a separation of lines on the *y*-axis. Figure 5*C* illustrates the commonalities across all six animals that are identified through averaging. For example, across animals, Pre-Frontal Cortex (PFC) generally dominated the low-frequency (<8 Hz) networks whereas the hippocampus generally dominated high-frequency networks between 80–150 Hz.

### Contributions of LFP versus multiunits

We next investigated the relative contribution of spikes and LFPs to the components. This was quantified as modality dominance (Zuure et al., 2020), which is the normalized difference between the root-mean-square of the LFP eigenvector elements and the root-mean-square of the multiunit eigenvector elements. A modality dominance value of zero indicates equal contribution of LFP and multiunits, whereas a value of one indicates no contribution of multiunits (a value of –1 would indicate no contribution of LFP channels).

The modality dominance values were close to one for all animals, recording sessions, and frequencies (Fig. 5*D*). This was not attributable to a difference in signal scaling between LFP and multiunits, because all time series signals were normalized to a mean of 0 and a variance of 1. However, normalizing to the first and second statistical moments does not preclude the possibility of differences in higher-order statistical characteristics. For example, the LFP channels had overall higher entropy (around 4 bits, averaged over all channels, animals, and experiment sessions) compared with the multiunits (1.7 bits on average; Fig. 5*E*).

On the other hand, it was not the case that multiunits made no contributions to the GED-identified networks. We re-ran the source separation for each frequency, excluding all multiunits from the dataset, and computed a *t* test at each frequency between the top eigenvalues from the multiunit-including and multiunit-excluding datasets. The difference was statistically significant (correcting for multiple comparisons using the false discovery rate method (Benjamini and Hochberg, 1995) for most frequencies except around 30–90 Hz (Fig. 5*F*).

Thus, the (Gaussian-smoothed) multiunits made a minor although statistically significant contribution to the matrix decomposition. This overall pattern is not surprising, considering that the LFP samples a larger volume and thus more neurons. On the other hand, there were more multiunit channels in the data matrix than LFP channels, and many of our multiunits may have reflected a combination of several neurons; thus, we interpret this finding to indicate that LFP signals are a richer source of information regarding cross-regional network formation than are action potentials.

### Within-frequency component dimensionality

The eigenvectors from the GED analysis carve out a low-dimensional subspace of narrowband activity, and we defined the dimensionality of that subspace as the number of eigenvalues that were larger than a significance threshold based on a null-hypothesis distribution of eigenvalues derived from permutation testing (Zuure et al., 2020).

**Figure 6.**
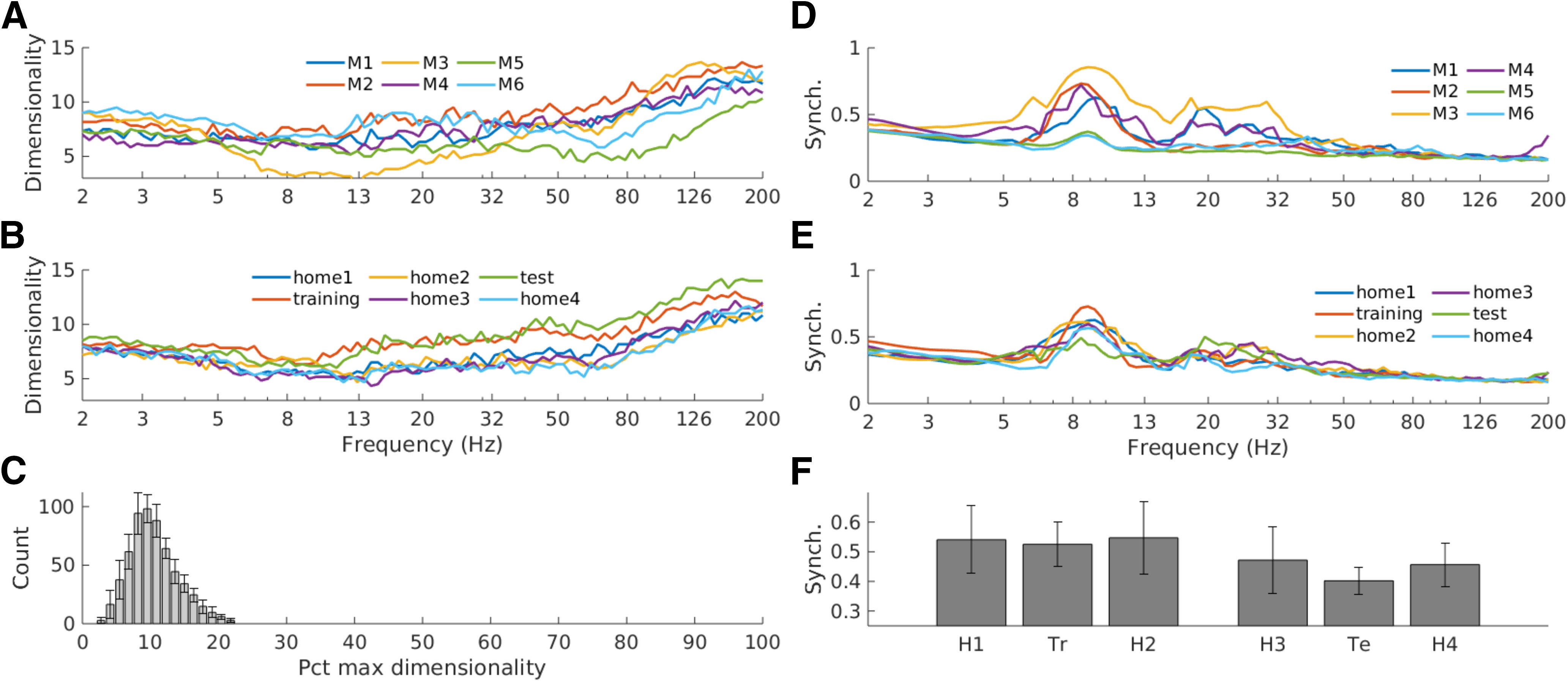
Generalized eigendecomposition reveals that narrowband subspaces are multidimensional (quantified as the number of statistically significant components), and components within each frequency are partially synchronized but non- redundant. ***A-B***, Subspace dimensionality across animals (***A***) and experiment sessions (***B***). ***C***, The distribution of all component dimensionalities, normalized to percent of the maximum possible dimensionality (the rank of the covariance matrices), revealed that the narrowband components spanned around 10% of the total possible signal dimensionality. ***D-F***, Phase synchronization between the top two components per frequency indicates both coordination and independence across within-frequency networks. Volume-conduction-independent phase synchronization tended to decline with frequency except for a prominent peak in theta/alpha (∼7-13 Hz) and a smaller prominence in beta ((∼15-30 Hz). The patterns were similar over different animals (***D***) and different sessions (***E***). ***F***, Average synchronization in the theta/alpha range for the different sessions.

The subspace dimensionality ranged from 2 to 16, and generally increased with higher frequencies (Fig. 6*A*,*B*). Higher dimensionality corresponds to the number of statistically separable networks operating at the same frequency. It is noteworthy that there is no pronounced “bump” in the theta range (∼4–10 Hz).

Note that this measure is not the total dimensionality of the signal; it is the dimensionality of the subspace that differentiates narrowband from broadband activity. Normalizing these raw numbers to the total dimensionality of the signal (assessed as the rank of the corresponding data covariance matrix) revealed that most narrowband subspaces occupied around 8–10% of the total signal space (Fig. 6*C*).

We investigated the dynamics within these subspaces by computing a volume-conduction-independent measure of phase synchronization (weighted phase lag index) between the top two components for each frequency and task session (note that GED eigenvectors are not constrained to orthogonality as with PCA, and thus within-frequency components can be correlated as long as they remain linearly separable). Synchronization strength varied between around 0.2 and 0.6 depending on the frequency, with strongest synchronization around theta and a smaller departure from the 1/f decay around the beta band (Fig. 6*D–F*). A repeated-measures ANOVA on session differences in the 7- to 12-Hz range indicated no main effect of task session (*F*_(5,25)_ = 1.3, *p* = 0.29).

## Discussion

In this study, we explored multivariate LFP and multiunit data from three brain regions in awake behaving mice using a combination of established and novel multivariate analysis methods to decompose the data into multiple spatial-spectral-temporal modes. We found that these were stable within each animal but variable across animals. These findings reveal a rich and multidimensional landscape of brain dynamics that highlight the complexity of on-going neural activity.

### Feature-guided source separation identifies large-scale narrowband networks

There are several dimension-reduction methods that are regularly applied in neuroscience, including PCA and ICA, factor analyses, and Tucker decompositions (Cunningham and Yu, 2014). It is often unclear which algorithms or which parameters are optimal (Cohen and Gulbinaite, 2014), and different algorithms can give similar or divergent results (Delorme et al., 2012; Cohen, 2017) depending on their maximization objectives.

GED has several advantages, including that it (1) separates narrowband from broadband activity while holding constant behavioral, cognitive, and other factors; (2) reduces the impact of artifacts or non-brain sources that have a relatively wide frequency distribution; (3) is amenable to inferential statistical thresholding, whereas other decompositions are descriptive and thus selecting components for subsequent interrogation may be subjective or biased; (4) takes into account both spatial and temporal dynamics instead of only spatial or only temporal features; (5) has higher signal-to-noise ratio characteristics and is more accurate at recovering ground truth simulations compared with PCA or ICA (Nikulin et al., 2011; de Cheveigné and Parra, 2014; Cohen, 2017; Zuure and Cohen, 2020).

An important finding here is the discovery that a single frequency band can group multiple distinct but spatially overlapping networks. In typical univariate or bivariate analyses, the LFP from a single electrode is treated as an independent statistical unit, based on the implicit assumption that the volume of tissue recorded by an electrode contains only one functional circuit. But a more likely scenario is that each electrode records a mixture of signals from multiple local circuits in the scale of hundreds of microns to a few mm, particularly in the presence of local coherence (Lindén et al., 2011). Thus, LFP is prone to the same kind of source mixing that affects MEG and EEG (Nunez and Srinivasan, 2006), although to a lesser extent. This, however, is fortuitous for multichannel recordings, because it means that linear separation methods that have been established in the EEG community are likely to be fruitful in invasive recordings.

The high reproducibility across sessions within each animal (Morrow et al., 2020), coupled with the low reproducibility across animals, suggests that the large-scale networks that manifest as coordinated LFP dynamics develop in idiosyncratic ways across different individuals. It is likely that at least some of the lower reproducibility across animals can be attributable to variability in surgical implantation and individual anatomy. On the other hand, aggregating data across animals based on a common *xyz* coordinate is standard practice in neuroscience, and our findings highlight the potential difficulties of this approach. Indeed, future research may gain more traction by combining data across individuals according to multivariate and functional/statistical properties, in addition to anatomic coordinates.

### The special role of theta in large-scale network formation

The theta frequency band, typically defined as 4–10 Hz in rodents in 4–8 Hz in humans, is widely implicated in a large range of cognitive processes, including spatial exploration, memory, motor function, and executive functioning. Clearly, there is no simple mapping of frequency band to cognitive process and indeed, even the same brain regions can generate multiple sources of theta independently (López-Madrona et al., 2020; Zuure et al., 2020), which may serve different cognitive functions (Töllner et al., 2017; Mikulovic et al., 2018). In the rodent brain, theta is most robust in the hippocampus, but also synchronizes with independent theta generators in the medial prefrontal cortex (O’Neill et al., 2013; Sigurdsson and Duvarci, 2015). Intracranial EEG studies in humans have confirmed that theta synchronization is widespread and linked to cognitive operations (Solomon et al., 2017).

The theta band stood out in many of our analyses, for example by having relatively strong within-frequency, cross-component synchronization (Fig. 6), sub-Gaussian kurtosis (Extended Data Fig. 2-1), and roughly equal contribution from all three regions (Fig. 5). Additionally, theta-band networks appeared to have the most anatomically consistent topographies across animals (see the small peak around theta in Fig. 4*A*). On the other hand, the subspace dimensionality of theta was not higher than other frequencies (Fig. 6*A*,*B*), suggesting that the theta is important for computational reasons, and is not simply the dominant frequency in general.

### LFP versus multiunit contributions to large-scale networks

It is perhaps unsurprising that the multiunits made relatively little statistical contribution to the narrowband components, considering that LFP samples a larger volume, has more signal complexity, and can be meaningfully separated into narrow frequency bands. On the other hand, the multiunits were recorded from the same electrodes, added unique information to the narrowband covariance matrices, and improved the overall separability of the narrowband components from broadband across most frequency ranges.

It is possible that LFP carries most of the inter-regional signaling (Yuste, 2015), considering that LFP reflects a multitude of intracellular and extracellular processes (Buzsáki et al., 2012; Reimann et al., 2013) that are modulated by population dynamics of excitatory and inhibitory cells (Mitzdorf, 1985). It is also possible that spikes carry important information that is spatiotemporally targeted and sparse, and therefore make contributions at a spatial scale smaller than what we investigated. Indeed, the eigendecomposition will prefer larger patterns of covariance over patterns driven by a single data channel. On the other hand, LFP is generally considered a proxy of the local input to a circuit while spikes are considered a proxy of the output of the circuit. Nonetheless, multiunits and LFP are rarely incorporated into the same data matrix as we have done, so their relative contributions should be quantitatively evaluated rather than intuitively inferred.

### Implications for novelty and memory

The main network characteristics we identified were stable across the task sessions. This seems to suggest that the weightings for combining the data channels, as defined by the GED, reflect stable neural architectures as opposed to transiently fluctuating cognitive states.

It is, however, possible that behavior modulates these network dynamics at a faster timescale than experiment sessions. Indeed, neural signatures of novelty processing may be transient, lasting only hundreds of milliseconds (Ranganath and Rainer, 2003) or tens of seconds when first introduced to a novel environment (França et al., 2014). For example, our camera tracking data (not reported here) revealed that animals tended to explore the objects for brief windows of time, sometimes only a few hundred milliseconds. These windows may have been too brief for sufficient neural network estimation, and because of the novelty of the data analysis methods, we chose to focus on characterizing the neural networks using maximal data to ensure high data quality. This could be explored in future studies by ensuring that a particular behavior is expressed for a longer period of time.

## Synthesis

Reviewing Editor: Arianna Maffei, SUNY Stony Brook Decisions are customarily a result of the Reviewing Editor and the peer reviewers coming together and discussing their recommendations until a consensus is reached. When revisions are invited, a fact-based synthesis statement explaining their decision and outlining what is needed to prepare a revision will be listed below. The following reviewer(s) agreed to reveal their identity: Mark Laubach.

Both reviewers found the study to be of interest as it presents the application of an advanced method for data analysis that has not been reported in this level of depth before. This approach is seen as particularly interesting as it allows for the analysis of high dimensional LFP/spike signals. The primary concerns were lack of details in some aspects of the analysis and data presentation and the need to provide clarifications for some aspects that were found to be ambiguous.

Reviewer #1

The manuscript reports a nice demonstration of the use of the generalized eigendecomposition, and provides new information on large-scale features of theta (4-10 Hz) activity. I had a few questions about the manuscript, specifically in the methods section. I hope that the paper will be more clear and useful to the community.

Comments

- In fairness, most studies of spike field coherence have been careful about looking at spikes and fields from the same electrode. This statement on page 3 should be revised: "...coherence between action potentials from one neuron with the LFP recorded on the same

electrode (Whittingstall K Logothetis, 2009)."

- Page 4: More details are needed on the stereotaxic coordinates. As written, "Each animal was implanted with 32 electrodes (see Figure 1a) spread across the prefrontal cortex (16 electrodes), parietal cortex (8 electrodes), and hippocampus (8 electrodes).", it is not possible to understand how the various areas were implanted. Stereotaxic coordinates for each craniotomy would be helpful. Also, were all electrodes placed directly into the brain, without any angles to the superficial layers? Doing so destroys connections via apical dendrites and should have major effects on the integrity of the neurons and would change how they signal.

- Page 4: The authors may have given away their identity in this statement: "Electrode design and surgeries are detailed elsewhere (van Hulten J. A. Cohen M. X, 2020)." In general, it would be best if they were to include all experimental details in the current manuscript.

- Page 4: "A metal reference screw was placed on the skull over the cerebellum." More details would be helpful about the placement of the reference. The area over the cerebellum is a challenge to place screws, given blood reservoirs below above that region of the brain. How deep were the screws placed and at what approximate locations? Do results vary with reference screw? For example, have the authors tried wrapping a ground wire around multiple ground screws, as is common in many labs? I think that this point should be expanded on in the paper, and if there are any data to comment on the effects of reference placement, it would be useful to have these in the paper.

- Page 5: "Components clearly identifiable as non-neural origins were projected out of the data." Could the authors say more about these components (in rebuttal and also paper)? This step adds a good bit of complexity to the data analysis workflow. Is it really necessary?

- Page 5: "Data from the first and last 10 seconds of each recording session were excluded from analyses." Why? Please explain in rebuttal and paper.

- Page 5-6: "(autoSort, available via our open code bitbucket.org/benglitz/controller-dnp/src/master/Access/SpikeSorting)" This link provides information on the authors. It should have been redacted prior to submission to eNeuro for purposes of review.

Reviewer #2

The authors apply multivariate methods to LFPs and multi-unit activity recorded from three (hippocampus, prefrontal and parietal cortices) regions of the mouse brain. Their analyses aim at revealing neural electrophysiological components with "multiple spatial, spectral, and temporal scales" in an unsupervised manner. In general, this is a highly interesting descriptive work which brings non-conventional tools to the field. This starts with the use of methods popular in the EEG community not usually seen in rodent LFP analysis; such as common average reference or ICA to remove muscle artefacts, for example. More importantly, the core method of the paper adds to the analysis repertoire for high dimensional datasets that are becoming more and more prevalent in e-phys studies. Therefore, I think this paper has a great merit of bringing this new approach to be discussed and developed by the community.

I have minor points mainly addressed to interpretation of the results.

1. The terminology used in the final paragraph of the discussion is confusing. What do the authors mean by "networks" in this context. From the sentence "Some networks (e.g., in theta) were spatially distributed across the brain, while other networks (typically in higher frequencies) were more localized to one or two regions." it seems "networks" is just referring to a frequency band. But elsewhere it seems they are referring to a component extracted by their method (which also has spatial information). I would use more direct terminology and avoid abstract terms such as "networks" in this context; even because the authors haven’t shown any evidence these components are reflecting "functional networks". If the authors want to hypothesize they are, then the wording has to be adjusted accordingly, i.e., by introducing these ideas as speculative.

2. Still in the same paragraph of the discussion, the authors state "The analyses revealed both idiosyncratic and reproducible network characteristics within- and across-animals, which suggests that the spatial organization of large-scale networks is subject to individual variability.". Unless I’m missing something, the interpretation given to the idiosyncratic components is far from being the most parsimonious. They are analysing LFPs recorded from fixed arrays aimed at highly complex structures. The authors say "anatomical targets of the electrode implants were identical in all animals" but they cannot guarantee that. Deviations as little as tenths of micrometers in the array location could change these components in the same mouse. Just as an example, even within the CA1 subfield of the hippocampus, the authors could be recording from radiatum or oriens layers, which are known to have different spectral characteristics; specially for oscillations faster than theta. Let alone other subfields as CA3 and dentate gyrus which by themselves have layers with different ephys properties. The same applies to deep vs superficial layers in the neocortex. In sum, claiming these non-reproducible patterns across mice as individual animal characteristics is extremely unconvincing. I recommend removing this claim or rewriting clearly state it as speculative offering the alternative explanations above.

3. " To generate this empirical null-hypothesis distribution, we randomly assigned each 2-second segment to average into the S or R covariance matrices.". I don’t understand what the authors are shuffling. Are they shuffling the 2-second window positions within channels to destroy actual correlations? Are they shuffling across S and R sets? Please be clearer here so the reader can have a precise sense of which features are being destroyed and kept in your shuffling procedure.

4. Please add numbers to your colorbars in Figure 2. By scaling your colorbars from minimum to maximum value will always show a contrast between channels, how do you know if that difference is not spurious? In an extreme scenario it could even be explained by the difference of impedances across electrodes. In the legend the authors wrote "color values are arbitrary"; but I think what they mean is that the scale/unit of the values is arbitrary. I’m concerned about this point because of the claim "...components reflected the coordination of multiple local dipoles (seen as the balance between blue and red colors in the map)". If the authors can’t address the real meaning and magnitude of such contrasts, they should turn down their claim. Just a suggestion: add a tick to the value 0 in the colorbar in Figure 2A (I’m guessing it is around green) as the absolute value of those values are the most important thing here. Also I’d consider using a divergent colormap.

5. " The average cluster center frequencies, along with their standard deviations, are shown in Figure 3c." The authors meant Figure 3d.

6. "These results show that grouping electrophysiology time series into spectral bands has an empirical basis and is not arbitrary or an artifact imposed by narrowband filtering.". Can the authors explain why the results show that? Your method necessarily produces an output, how given the one you have shows the bands are not arbitrary or artifactual? What kind of result would show you otherwise? In other words, how would those claims be falsifiable?

7. The authors list advantages of the GED method in comparison to other approaches such as ICA and PCA. However, the authors found a stable global theta component, which is known for decades; and a number of higher frequency components that are not reproducible across mice. Then, they fail to show differences of the components in behavior. So, in the spite of the theoretical advantages claimed, the method did not reveal novel information from these recordings. Not even the beta2 oscillations cited by the authors that dominate the hippocampus during the first tens of seconds of novelty seems to be picked up by the method. In sum, the advantages of the method weren’t shown to provide advances to the field. I understand this could be out of the scope of this present work, but I would suggest the authors at least discuss in more depth the possibilities of follow up analyses. For example, the authors have never used the instantaneous speed of the animals to control for potential effects in test vs home cage sessions. Also, how exactly the authors would use their method to incorporate time for the analyses, for example to extract beta2 during novelty?
