# Supplementary material for: Large-Scale and Multiscale Networks in the Rodent Brain during Novelty Exploration
Source: eNeuro. 2021 May 8;8(3):ENEURO.0494-20.2021. doi: 10.1523/ENEURO.0494-20.2021 (PMC8121262; doi:10.1523/ENEURO.0494-20.2021)
Supplement: Extended Data Figure 1-1 — Left plot shows an example multiunit correlation matrix from one recording session. The right plot shows a histogram of all unique off-diagonal correlation values. These plots illustrate that our spike-sorting approach was not overly contaminated by identifying the same units on multiple channels. Download Figure 1-1, PDF file. [file enu-eN-NWR-0494-20-s01.pdf]

Large- and multi-scale networks in the rodent brain during novelty exploration

Michael X Cohen[1,*], Bernhard Englitz[2], Arthur S C França[1]

[1] Radboud University Medical Center, Donders Centre for Medical Neuroscience

[2] Computational Neuroscience Lab, Department of Neurophysiology, Donders Institute for Brain, Cognition and Behavior, Radboud University Nijmegen, The Netherlands

* correspondence: mikexcohen@gmail.com

Funding: MXC is funded by an ERC-StG 638589 and a Hypatia fellowship from the Radboud University Medical Center. AF is funded by the ERC. BE is supported by a NWO VIDI Grant (016.189.052) and a NWO ALW Open Grant (ALWOP.346).

Competing or conflicting interests: none

Acknowledgements: We thank Mihaela Gerova for assistance with data cleaning and preparation.

Keywords: Local field potential, neural oscillations, novelty, prefrontal cortex, parietal cortex, hippocampus, large-scale networks, eigendecomposition, source separation.

Running title: Large multiscale networks in the rodent brain
