# Supplementary material for: Large-Scale and Multiscale Networks in the Rodent Brain during Novelty Exploration
Source: eNeuro. 2021 May 8;8(3):ENEURO.0494-20.2021. doi: 10.1523/ENEURO.0494-20.2021 (PMC8121262; doi:10.1523/ENEURO.0494-20.2021)
Supplement: Extended Data Figure 2-1 — Kurtosis, a measure of non-Gaussianity of a distribution (see text below), computed on frequency-specific component time series. The red and blue lines in panel A show kurtosis per frequency for the narrowband-filtered time series (blue) and amplitude envelope (red), averaged over all animals and sessions. The horizontal dashed line indicates the expected kurtosis of a pure Gaussian distribution. B, Kurtosis over frequencies for each animal separately. Note the striking decrease in kurtosis in the theta band in all animals. C, Example time series histograms illustrating the platykurtic effect at 8 and 11 Hz for two different animals and sessions. Distribution shape via kurtosis. Non-Gaussianity is considered an indicator of an information-rich signal. This comes from the central limit theorem, which leads to the assumption that random noise, and random linear mixtures of signals, will produce Gaussian distributions. We therefore quantified the kurtosis (4th statistical moment of a distribution; the kurtosis of a pure Gaussian distribution is 3) as a measure of the non-Gaussianity of the component time series. We computed kurtosis for the narrowband filtered signal and its amplitude envelope at each component. Component time series kurtosis was computed as the 4th statistical moment of the component time series. We extracted kurtosis from both the real part of the narrowband signal and the amplitude envelope (extracted via the Hilbert transform). The amplitude envelope had overall higher kurtosis (Extended Data Fig. 2-1), which is not surprising considering that amplitude is a strictly non-negative quantity. Nearly all frequencies had kurtosis higher than 3, indicating leptokurtic distributions characterized by narrow peaks and fatter tails. This is consistent with suggestions that brain activity is characterized by extreme events and long-tailed distributions (Buzsáki and Mizuseki, 2014). Curiously, all six animals exhibited a dip in kurtosis in the theta band (∼9 H [file enu-eN-NWR-0494-20-s02.docx]

**Large- and multi-scale networks in the rodent brain during novelty exploration**

**Extended data**


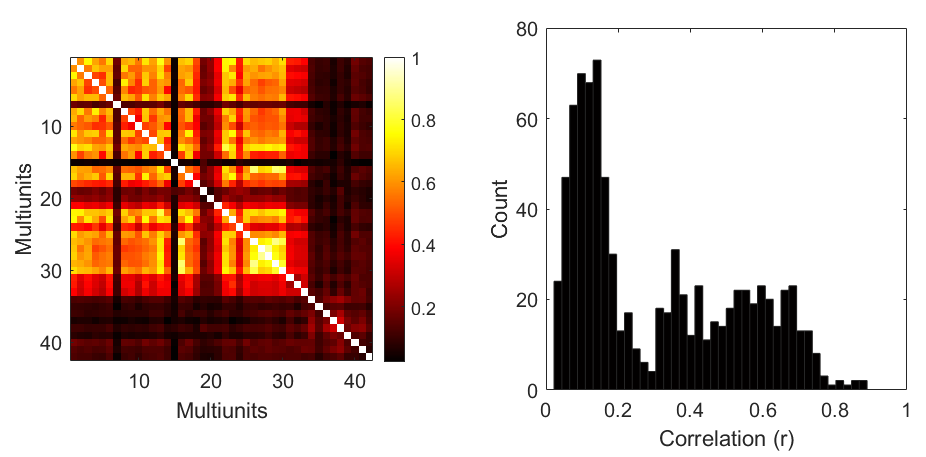


***Figure 1-1****. Left plot shows an example multiunit correlation matrix from one recording session. The right plot shows a histogram of all unique off-diagonal correlation values. These plots illustrate that our spike-sorting approach was not overly contaminated by identifying the same units on multiple channels.*


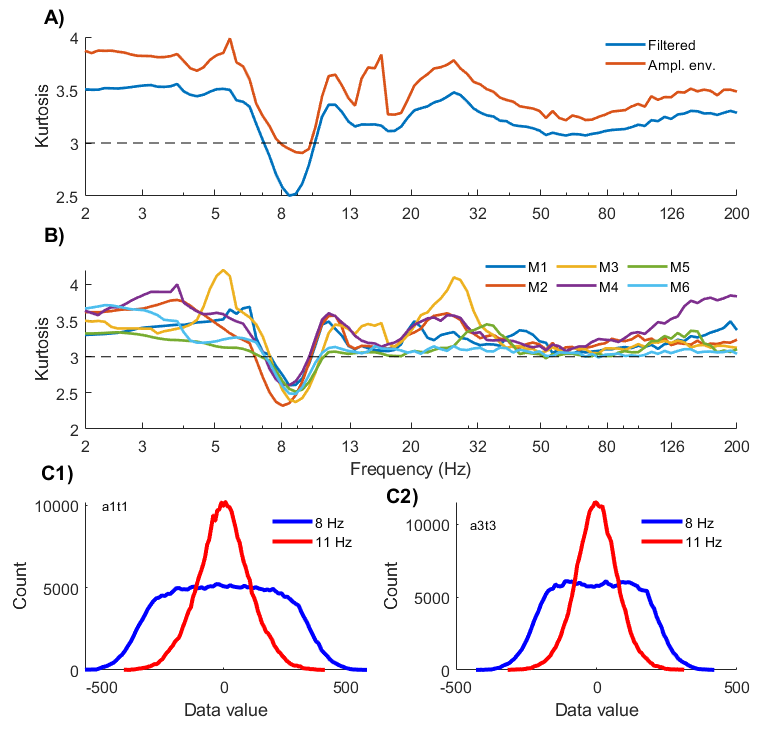


***Figure 2-1****. Kurtosis, a measure of non-Gaussianity of a distribution (see text below), computed on frequency-specific component time series. The red and blue lines in panel A show kurtosis per frequency for the narrowband-filtered time series (blue) and amplitude envelope (red), averaged over all animals and sessions. The horizontal dashed line indicates the expected kurtosis of a pure Gaussian distribution. B) Kurtosis over frequencies for each animal separately. Note the striking decrease in kurtosis in the theta band in all animals. C) Example time series histograms illustrating the platykurtic effect at 8 Hz and 11 Hz for two different animals and sessions.*

*Distribution shape via kurtosis*. Non-Gaussianity is considered an indicator of an information-rich signal. This comes from the central limit theorem, which leads to the assumption that random noise, and random linear mixtures of signals, will produce Gaussian distributions. We therefore quantified the kurtosis (4th statistical moment of a distribution; the kurtosis of a pure Gaussian distribution is 3) as a measure of the non-Gaussianity of the component time series. We computed kurtosis for the narrowband filtered signal and its amplitude envelope at each component.

Component time series kurtosis was computed as the 4th statistical moment of the component time series. We extracted kurtosis from both the real part of the narrowband signal and the amplitude envelope (extracted via the Hilbert transform). The amplitude envelope had overall higher kurtosis (Figure 2-1), which is not surprising considering that amplitude is a strictly non-negative quantity.

Nearly all frequencies had kurtosis higher than 3, indicating leptokurtic distributions characterized by narrow peaks and fatter tails. This is consistent with suggestions that brain activity is characterized by extreme events and long-tailed distributions [(Buzsáki and Mizuseki, 2014)](https://paperpile.com/c/8mKmQY/cD4U2). Curiously, all six animals exhibited a dip in kurtosis in the theta band (~9 Hz) (Figure 2-1b), indicating a platykurtic distribution with data values clustered towards zero and relatively fewer data points having extreme values (the tails of the distributions) (Figure 2-1c). This may be related to the known sawtooth-like shape of hippocampal theta [(Scheffer-Teixeira and Tort, 2016)](https://paperpile.com/c/8mKmQY/4RH7Q).

Note that unlike independent components analysis, GED is based purely on the signal covariance (second moment) and not on any higher-order statistical moments. Thus, non-Gaussian distributions are not trivially imposed by the decomposition method, but instead arose from the data without bias or selection.

[Buzsáki G, Anastassiou CA, Koch C (2012) The origin of extracellular fields and currents — EEG, ECoG, LFP and spikes. Nat Rev Neurosci 13:407.](http://paperpile.com/b/8mKmQY/3PvJD)

[Scheffer-Teixeira R, Tort AB (2016) On cross-frequency phase-phase coupling between theta and gamma oscillations in the hippocampus. Elife 5.](http://paperpile.com/b/8mKmQY/4RH7Q)
